# 17*β*-Estradiol Increases APE1/Ref-1 Secretion in Vascular Endothelial Cells and Ovariectomized Mice: Involvement of Calcium-Dependent Exosome Pathway

**DOI:** 10.3390/biomedicines9081040

**Published:** 2021-08-18

**Authors:** Yu-Ran Lee, Hee-Kyoung Joo, Eun-Ok Lee, Sungmin Kim, Hao Jin, Yeon-Hee Choi, Cuk-Seong Kim, Byeong-Hwa Jeon

**Affiliations:** 1 Research Institute for Medical Sciences, College of Medicine, Chungnam National University, 266 Munhwa-ro, Jung-gu, Daejeon 35015, Korea; lyr0913@cnu.ac.kr (Y.-R.L.); hkjoo79@cnu.ac.kr (H.-K.J.); y21c486@naver.com (E.-O.L.); s13845@naver.com (S.K.); jinhao0508@gmail.com (H.J.); yeonhee970@gmail.com (Y.-H.C.); cskim@cnu.ac.kr (C.-S.K.); 2 Department of Physiology, College of Medicine, Chungnam National University, 266 Munhwa-ro, Jung-gu, Daejeon 35015, Korea

**Keywords:** apurinic/apyrimidinic endonuclease-1/redox factor-1, 17β-estradiol, estrogen receptor, endothelial cells, calcium, exosome

## Abstract

Apurinic/apyrimidinic endonuclease-1/redox factor-1 (APE1/Ref-1) is a multifunctional protein that can be secreted, and recently suggested as new biomarker for vascular inflammation. However, the endogenous hormones for APE1/Ref-1 secretion and its underlying mechanisms are not defined. Here, the effect of twelve endogenous hormones on APE1/Ref-1 secretion was screened in cultured vascular endothelial cells. The endogenous hormones that significantly increased APE1/Ref-1 secretion was 17β-estradiol (E2), 5𝛼-dihydrotestosterone, progesterone, insulin, and insulin-like growth factor. The most potent hormone inducing APE1/Ref-1 secretion was E2, which in cultured endothelial cells, E2 for 24 h increased APE1/Ref-1 secretion level of 4.56 ± 1.16 ng/mL, compared to a basal secretion level of 0.09 ± 0.02 ng/mL. Among the estrogens, only E2 increased APE1/Ref-1 secretion, not estrone and estriol. Blood APE1/Ref-1 concentrations decreased in ovariectomized (OVX) mice but were significantly increased by the replacement of E2 (0.39 ± 0.09 ng/mL for OVX vs. 4.67 ± 0.53 ng/mL for OVX + E2). E2-induced APE1/Ref-1secretion was remarkably suppressed by the estrogen receptor (ER) blocker fulvestrant and intracellular Ca^2+^ chelator 1,2-Bis(2-aminophenoxy)ethane-N,N,N′,N′-tetraacetic acid tetrakis (acetoxymethyl ester) (BAPTA-AM), suggesting E2-induced APE1/Ref-1 secretion was dependent on ER and intracellular calcium. E2-induced APE1/Ref-1 secretion was significantly inhibited by exosome inhibitor GW4869. Furthermore, APE1/Ref-1 level in CD63-positive exosome were increased by E2. Finally, fluorescence imaging data showed that APE1/Ref-1 co-localized with CD63-labled exosome in the cytoplasm of cells upon E2 treatment. Taken together, E2 was the most potent hormone for APE1/Ref-1 secretion, which appeared to occur through exosomes that were dependent on ER and intracellular Ca^2+^. Furthermore, hormonal effects should be considered when analyzing biomarkers for vascular inflammation.

## 1. Introduction

Apurinic/apyrimidinic endonuclease 1/redox factor-1 (APE1/Ref-1) is a multifunctional protein suggested as a new biomarker of vascular inflammation [1,2]. Basically, APE1/Ref-1 plays roles in transcriptional regulation through redox modification and base excision repair [3]. Since it was first reported in 2013 that APE1/Ref-1 secretion is increased by intracellular hyperacetylation [4], it has subsequently been reported that its secretion is increased in lipopolysaccharide-induced endotoxemic animal models [5], apolipoprotein E-deficient mice fed Western-type diets [1], and patients with coronary artery disease [6], suggesting its usefulness as a new biomarker for vascular inflammation.

Cell signaling are affected by a variety of stimulators that are responsible for the secretion of proteins in cells [7]. Hormone secretion is involved in maintaining homeostasis in vivo. Changes in specific protein secretion can play an important role in the diagnosis or prognosis of various diseases, such as systemic inflammation [8]. The presence of APE1/Ref-1 in the extracellular environment as a biomarker has been suggested as it is actively secreted in specific diseases, and it may also be used as evidence of non-specific tissue damage since it can be released from cells upon cell death. Identifying substances that increase the secretion of proteins without causing cell death could help in the development of new biomarkers for specific conditions or diseases. The secreted substances in the response to hormone have been hypothesized to play an important role in the regulation of vascular inflammation.

The biological functions of secreted APE1/Ref-1 are uncovering. Extracellular APE1/Ref-1 is known to have its own function and can affect surrounding and distant cells. Enzymatically active APE1/Ref-1 protein that functions as an endonuclease is secreted in response to genotoxic stress [9]. Secretory APE1/Ref-1 has been reported to have a role in the inhibition of vascular inflammation via thiol-disulfide exchange in the tumor necrosis factor (TNF) receptor [10]. Therefore, the secreted APE1/Ref-1 might have anti-inflammatory properties and the redox activity of its cysteine residue has recently been associated with its anti-inflammatory activity in vivo in animal models [11].

The subcellular location of APE1/Ref-1 is regulated by post-transcriptional modifications, including acetylation. The histone deacetylase inhibitor trichostatin A induces APE1/Ref-1 secretion in human embryonic kidney (HEK) 293T cells and mutations in APE1/Ref-1 at lysine residues 6 and 7 (K6R/K7R) markedly diminish its secretion [4]. Secretion of APE1/Ref-1 is observed in response to hyperacetylation in triple-negative breast cancer cell lines, resulting in significantly decreased cell viability and the induction of apoptosis [12]. In addition, nitrosylation selectively induces cytosolic translocation of APE1/Ref-1 with Cys93 and Cys310 being critical for the nitrosylation-mediated cytosolic translocation [13].

Protein secretion may occur through either the classical or non-classical secretory pathway. Proteins secreted through the classical pathway typically contain N-terminal signal peptides that direct the proteins to the translocation apparatus of the endoplasmic reticulum [14]. The mechanisms of APE1/Ref-1 secretion have not yet been fully elucidated. Some reports on the pathway of APE1/Ref-1 secretion suggested that it may be secreted via exosomes [9,12,15] or the ATP-binding cassette transporter [16].

Finding out which endogenous hormone can affect the secretion of APE1/Ref-1 can be utilized to understand the pathophysiology of hormone imbalance in vascular inflammation. Therefore, the current study aimed to identify potential endogenous hormones that increase APE1/Ref-1 secretion in vascular endothelial cells under conditions that do not induce cell death; it also aimed to reveal the underlying secretion mechanism.

## 2. Materials and Methods

### 2.1. Cell Culture and Reagents

Human umbilical vein endothelial cells (HUVECs) (C2517A, Lonza, Walkersville, MD, USA) were cultured in endothelial growth medium (EGM-2) purchased from Lonza Bioscience (Walkersville, MD, USA). The HUVECs were maintained in a humidified atmosphere of 95% air and 5% CO_2_ at 37 °C. Norepinephrine, acetylcholine, triiodothyronine (T3), thyroxine (T4), insulin-like growth factor (IGF), cortisol, aldosterone, insulin, glucagon, 5α-dihydrotestosterone (DHT), 17β-estradiol (E2), progesterone (P4), estrone (E1), estriol (E3), fulvestrant, N(ω)-nitro-L-arginine methyl ester (L-NAME), 1,2-Bis(2-aminophenoxy)ethane-N,N,N′,N′-tetraacetic acid tetrakis(acetoxymethyl ester) (BAPTA-AM), GW4869, and dimethyl sulfoxide (DMSO) were purchased from Sigma Aldrich (St. Louis, MO, USA). An ExoQuick-Tc exosome isolation kit was purchased from System Biosciences (Palo Alto, CA, USA). The polyclonal antibody against APE1/Ref-1 was obtained from MediRedox, Inc. (Daejeon, Korea) and, the anti-CD63 antibody was obtained from Biobyt (Cambridge, UK). The monoclonal antibody against anti-heat shock protein-70 (HSP70, C92F3A-5) was obtained from Enzo Life Science (Farmingdale, NY, USA), the anti-CD9 (C-4) antibody was obtained from Santa Cruz Biotechnology (Dallas, TX, USA) and anti-ALG-2 interacting protein X (Alix, 3A9) antibody was obtained from Thermo-Fisher Scientific Inc (Waltham, MA, USA).

### 2.2. Cell Viability Assay Using Reducing Potentials of Cells

The viability and cytotoxicity of HUVECs were analyzed using a RealTime-Glo™ MT cell viability assay kit (Promega, Madison, WI, USA), according to the manufacturer’s instructions. Briefly, HUVECs were plated into 96-well white cell culture plates at a density of 5 × 10^3^ cells/well. After 24 h of incubation, the pro-substrate and luciferase were added at the same time as that of the hormones to continuously monitor the viability of the HUVECs in real-time. Luminescence intensity at the desired time points was measured using a Glo-Max™ multimode reader (Promega, Madison, WI, USA).

### 2.3. Quantification of Secretory APE1/Ref-1

Mouse plasma and cell culture media were centrifuged at 3000 rpm for 10 min to obtain cell-free samples as previously reported [11]. Secreted APE1/Ref-1 levels were determined using a APE1/Ref-1 sandwich enzyme-linked immunosorbent assay (ELISA) kit (MediRedox, Inc., Daejeon, Korea) according to the manufacturer’s instructions. Secreted APE1/Ref-1 levels (ng/mL for plasma and ng/10^5^ cells for supernatants) were calculated against a standard curve generated using recombinant human APE1/Ref-1 protein (MediRedox, Inc., Daejeon, Korea).

### 2.4. Isolation of Exosome in Cell Culture Media

To isolate the exosome from the cell culture medium of HUVECs, the medium was changed to medium without fetal bovine serum. The HUVECs were then treated with E2 for 24 h and the cell culture medium was collected and centrifuged at 500× *g* for 10 min. The clarified supernatant was collected, and debris and vesicle over 0.22 µm in diameter was removed through filtration using a 0.22 µm syringe filter (Millipore, Billerica, MA). exosomes were isolated using an Exoquick-TC isolation kit (System Biosciences, Palo Alto, CA, USA) that precipitates exosomes based on polyethylene glycol precipitation as recommended by the manufacturer. The isolated exosomes were confirmed using immunoblotting for CD63, CD9, HSP70, and Alix.

### 2.5. Establishment of an Ovariectomized Mice Model

Animal experiments were performed using female C57BL/6J mice, 7–8 weeks of age (DooYeol Biotech, Seoul, Korea). The animal protocol was approved by the Ethics Committee of Animal Experimentation of Chungnam National University Hospital (CNUH-017-A0025) and all experiments were performed in accordance with the Guide for the Care of Use of Laboratory Animals published by the US National Institutes of Health (NIH Publication, 8th edition, 2011). All surgeries and pump implantations were performed using aseptic procedures.

All mice in the ovariectomized (OVX) group underwent bilateral ovariectomy [17] using a single ventral approach. The sham group suffered the same surgery except that their ovaries were preserved. The OVX and sham mice were randomly divided into two groups for implantation of Alzet osmotic minipumps (model number 1002, for 14-day delivery at 0.26 μL/hour, Durect Corp, Cupertino, CA, USA). The osmotic pumps with E2 were implanted subcutaneously between the scapulae via a small incision. E2 was dissolved in 10% dimethyl sulfoxide (DMSO; Sigma-Aldrich, St. Louis, MO, USA). The concentration of the experimental agent was 35 μg/mL, which resulted in a delivery rate of approximately 0.25 μg/day.

### 2.6. Immunoblot Analysis

Proteins were separated by 10% sodium dodecyl sulphate–polyacrylamide gel electrophoresis (SDS-PAGE) and transferred to a polyvinylidene fluoride (PVDF) membranes. After blocking with 5% non-fat dry milk in tris-buffered saline (TBS) containing 0.05% Tween 20, the membranes were incubated with primary antibodies anti-APE1/Ref-1, anti-CD63, anti-CD9, anti-HSP70, and anti-Alix for 18 h at 4 °C. The membranes were then treated with an appropriate horseradish peroxidase (HRP)-conjugated secondary antibody and the chemiluminescent signal was developed using Super Signal West Pico or Femto Substrate (Pierce Biotechnology, Rockford, IL, USA).

### 2.7. Immunofluorescence

HUVECs were seeded on glass coverslips and transiently transfected with plasmids *pEGFP-APE1/Ref-1* [18] or *pCT-RFP-CD63* (System Biosciences, Palo Alto, CA, USA) using Effectene transfection reagent (QIAGEN Inc., Santa Clarita, CA, USA). Cells were fixed with 4% paraformaldehyde and the stained with 4′,6-diamidino-2-phenylindole (DAPI) for 3 min. The coverslips were mounted onto microscope slides using a fluorescence mounting solution and the signals visualized using a confocal microscope (Leica Microsystems, Buffalo Grove, IL, USA).

### 2.8. Statistics

All data are presented as the mean ± SEM of at least three independent biological replicates unless stated otherwise. The statistical tests used are indicated in the respective figure legends. For all tests, the following *p*-values were applied: *** *p* < 0.001, ** *p* < 0.01, * *p* < 0.05, and not significant (ns) *p* > 0.05. All statistical analyses were performed using GraphPad Prism 9 for the Mac OS (GraphPad Software Inc., La Jolla, CA, USA).

## 3. Results

### 3.1. Identification of Hormones that Induce APE1/Ref-1 Secretion in HUVECs

We first investigated endogenous hormones that could induce APE1/Ref-1 secretion without changing cell viability. Twelve representative endogenous hormones were selected and screened for their ability to increase the release of APE1/Ref-1 in vascular endothelial cells. The hormones and their concentrations used in this study are shown in Table 1. APE1/Ref-1 concentrations in the cell culture media were measured using an APE1/Ref-1 sandwich ELISA technique. Cell viability was measured after 3 h and 24 h of treatment using a real-time GLO luminescence technique that measures cell metabolism and was reported as the ratio of luminescence change relative to the amount of luminescence before hormone administration.

**Figure 1:**
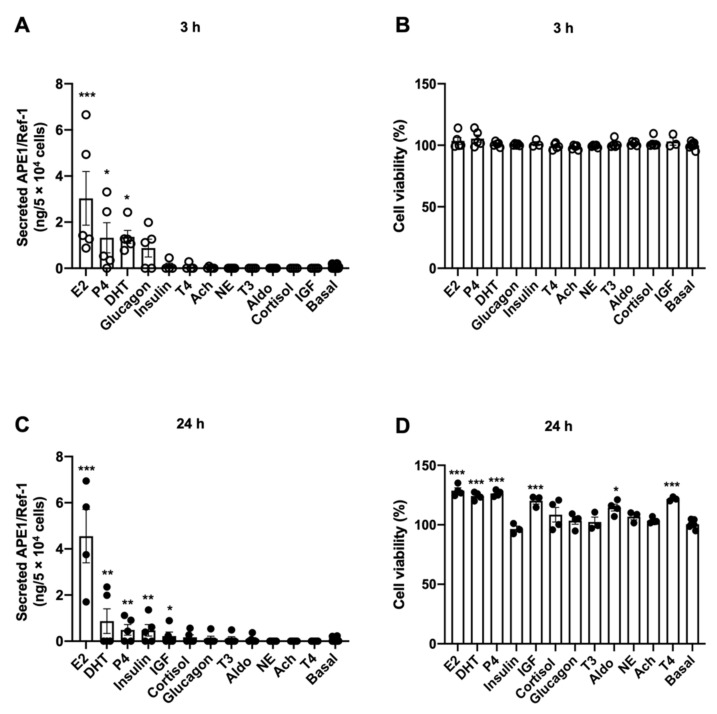
Identification of endogenous hormone for induction of apurinic/apyrimidinic endonuclease-1/redox factor-1 (APE1/Ref-1) secretion. (**A**) Effect of short-term (3 h) treatment of hormones on APE1/Ref-1 levels in the culture media of vascular endothelial cells. (**B**) Effect of short-term (3 h) treatment of hormones on cell viability of vascular endothelial cells. (**C**) Effect of long-term (24 h) treatment of hormones on APE1/Ref-1 levels in the culture media of vascular endothelial cells. (**D**) Effect of long-term (24 h) treatment of hormones on cell viability of vascular endothelial cells. Vascular endothelial cells were treated with each hormone for 3 h or 24 h. APE1/Ref-1 levels in culture supernatant were measured using an APE1/Ref-1 sandwich ELISA assay as describe Material and Methods. Columns, mean (n = 4–5); dot plot, SE. *** *p* < 0.001, ** *p* < 0.01, * *p* < 0.05 indicates a significant difference compared to the control cells (Basal) according to an unpaired *t*-test.

The concentrations of APE1/Ref-1 in the cell culture media of HUVECs at rest for 3 h and 24 h were 0.07 ± 0.02 ng/mL and 0.09 ± 0.02 ng/mL (n = 5–6), respectively. The change in APE1/Ref-1 concentration in the cell culture media was analyzed after treatment with each hormone for 3 h. The hormones that induced significant increases in APE1/Ref-1 concentrations were E2 (3.03 ± 1.16 ng/mL), DHT (1.36 ± 0.28 ng/mL), P4 (1.33 ± 0.65 ng/mL), and glucagon (0.875 ± 0.38 ng/mL) (Figure 1A). As expected, treatment of the HUVECs with the selected hormones for 3 h did not affect cell viability (Figure 1B). The changes in APE1/Ref-1 concentrations in the cell culture media were also analyzed after long-term treatment of the HUVECs with each hormone for 24 h. The endogenous hormones that induced significant increases in the APE1/Ref-1 concentrations were E2 (4.56 ± 1.16 ng/mL), DHT (0.87 ± 0.53 ng/mL), P4 (0.492 ± 0.23 ng/mL), insulin (0.47 ± 0.25 ng/mL), and IGF (0.23 ± 0.17 ng/mL) (Figure 1C). None of the selected 12 hormones decreased cell viability of the HUVECs after 24 h of treatment compared with that of the control group (Figure 1D). Taken together, among 12 endogenous hormones, E2, DHT, P4, insulin, and IGF (in descending order) increased APE1/Ref-1 secretion without decreasing cell viability. Interestingly, the most potent endogenous hormone for APE1/Ref-1 secretion in vascular endothelial cells was an estrogen.

### 3.2. 17β-Estradiol Induced APE1/Ref-1 Secretion in HUVECs

**Figure 2:**
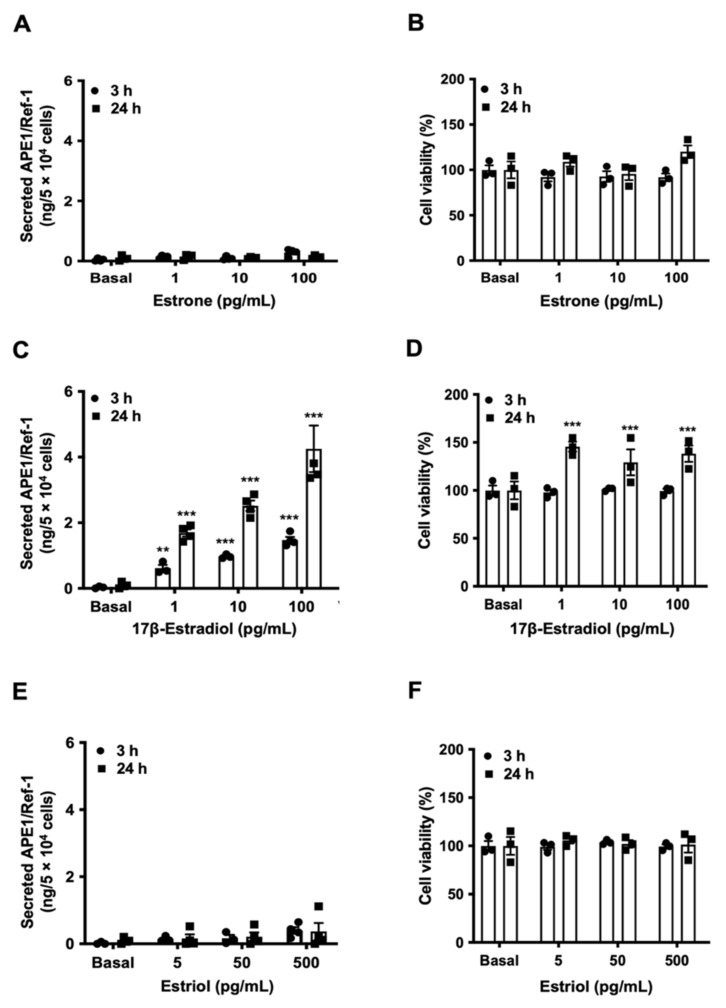
Differential regulation of apurinic/apyrimidinic endonuclease-1/redox factor-1 (APE1/Ref-1) secretion by estrogen. The effect of estrone (**A**), 17β-estradiol (**C**), and estriol (**E**) on APE1/Ref-1 levels in the culture media of vascular endothelial cells. The effect of estrone (**B**), 17β -estradiol (**D**), and estriol (**F**) on cell viability of vascular endothelial cells. Columns, mean (n s= 3–4); dot plot, SE. *** *p* < 0.001, ** *p* < 0.01, indicates a significant difference compared to that of control cells (Basal) according to an unpaired *t*-test.

Estrogen is known to exist as three main types: estrone (E1), 17β-estradiol (E2), and estriol (E3). Next, we investigated which type(s) of estrogen was able to induce APE/1Ref-1 secretion in the endothelial cells. To evaluate the effect of three types of estrogen on APE1/Ref-1 secretion, APE1/Ref-1 levels were measured after 3 h and 24 h of treatment in various dosage of the estrogens. E1-treated or E3-treated HUVECs did not demonstrate the ability to induce APE1/Ref-1 secretion (Figure 2A,E). Treatment with E1 and E3 for 3 h did not affect cell viability of the vascular endothelial cells (Figure 2B,F). However, when HUVECs were treated with E2, APE1/Ref-1 secretion was increased in a dose-dependent manner (Figure 2C). E2 treatment for 24 h induced an increase in cell viability, suggesting cell growth (Figure 2D). Therefore, we confirmed that E2 is a hormone to increase APE1/Ref-1 secretion without causing cell death.

### 3.3. 17β-Estradiol Increased APE1/Ref-1 Secretion in Ovariectomized (OVX) Mice

**Figure 3:**
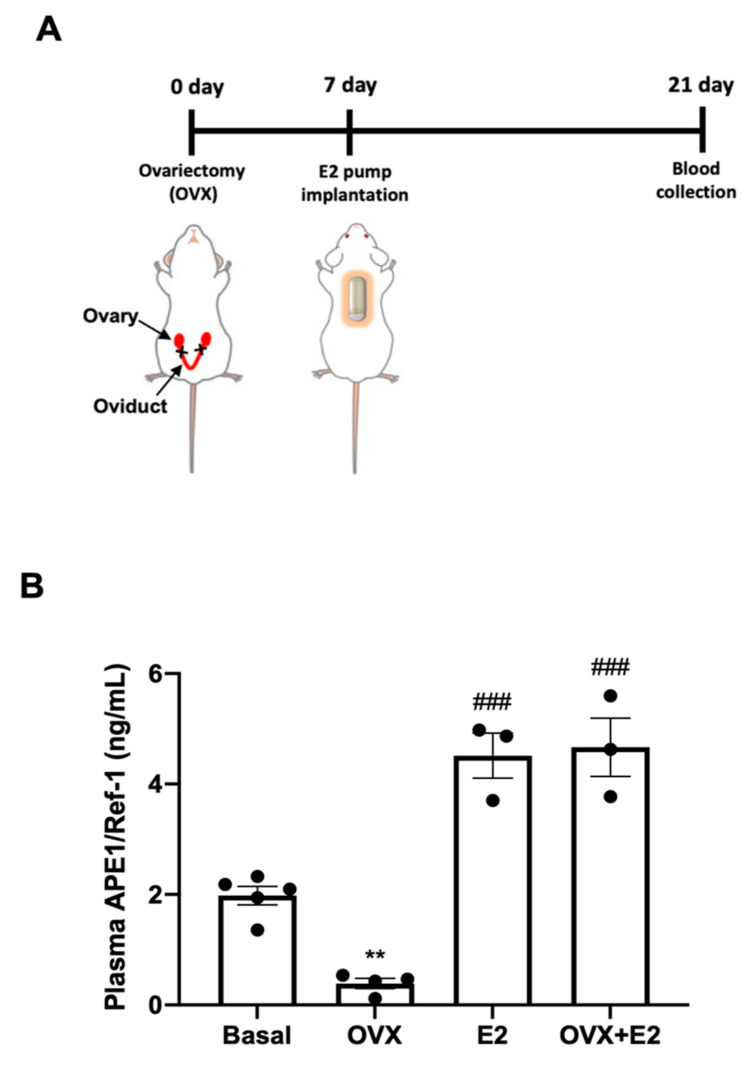
17β-Estradiol (E2) increases plasma apurinic/apyrimidinic endonuclease-1/redox factor-1 (APE1/Ref-1) levels in ovariectomized (OVX) mice. (**A**) Experimental schedule for 17β-estradiol treatment and performing the APE1/Ref-1 assay. Ovariectomy (OVX) was performed 7 d prior to the implantation of the E2 pump in mice. APE1/Ref-1 blood levels were analyzed using an APE1/Ref-1 sandwich enzyme-linked immunosorbent assay (ELISA) 14 d after implantation of the E2 pump. (**B**) Effect of E2 implantation on plasma APE1/Ref-1 levels in ovariectomized mice. Basal group, sham OVX surgery with 10% dimethyl sulfoxide (DMSO) in the osmotic pump; OVX group, ovariectomized mice with 10% DMSO in the osmotic pump; E2 group, sham OVX surgery with E2 in the osmotic pump (10 µg/kg/day); OVX + E2 group, ovariectomy with E2 in the osmotic pump (10 µg/kg/day). Columns, mean (n = 3–4 animals per group.); dot plot, SE. ** *p* < 0.05 vs. the Basal group and ### *p* < 0.001 vs. the OVX group based on one-way ANOVA analysis followed by Bonferroni’s multiple comparison test.

To extend the novel concept of 17β-estradiol (E2) as an inducer of APE1/Ref-1 secretion in cultured endothelial cells, we attempted to determine whether it could increase the levels of plasma APE1/Ref-1 in OVX mice. Figure 3A shows the experimental design for the evaluation of the role of estradiol in OVX mice. The effect of E2 on APE1/Ref-1 concentration in the blood was evaluated 14 days after insertion of the E2-containing osmotic pump. As shown in Figure 3B, plasma APE1/Ref-1 levels in basal conditions and OVX mice were 1.98 ± 0.17 ng/mL and 0.39 ± 0.09 ng/mL, respectively. These results are shown that the plasma APE1/Ref-1 levels were significantly reduced by the removal of ovary, suggesting a physiological role of the ovary in regulating blood APE1/Ref-1 concentrations in vivo. The replacement of E2 for 14 days increased plasma APE1/Ref-1 levels both normal and OVX mice (4.51 ± 0.41 ng/mL and 4.67 ± 0.53 ng/mL, respectively), compared with OVX mice (0.39 ± 0.09 ng/mL). Taken together, these findings confirmed that estrogen in ovary is important hormone for the in vivo regulation of blood APE1/Ref-1, and administration of E2 can increases the concentration of APE1/Ref-1 in the blood.

### 3.4. 17β-Estradiol-Induced APE1/Ref-1 Secretion Depend on the Binding of ER and Intracellular Calcium

**Figure 4:**
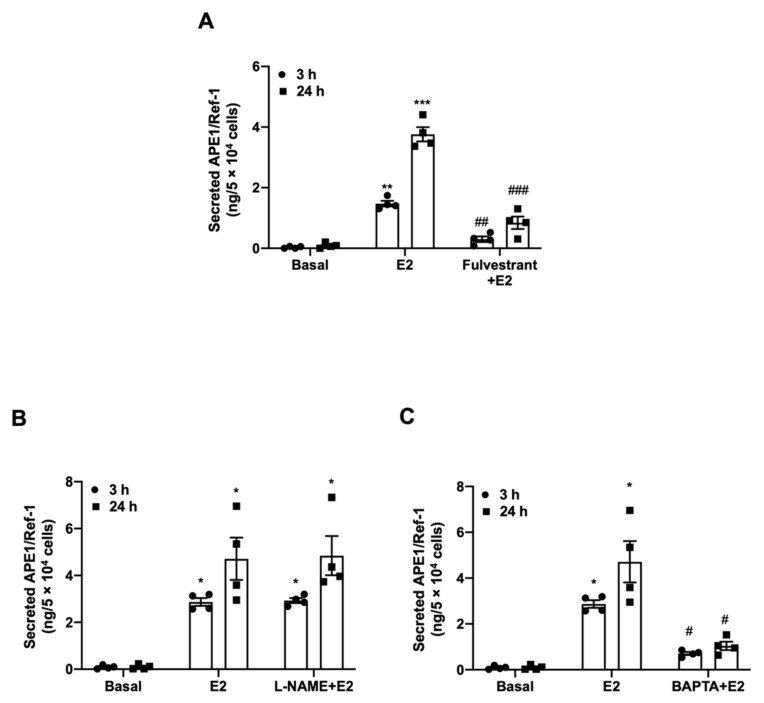
17β-estradiol (E2)-induced apurinic/apyrimidinic endonuclease-1/redox factor-1 (APE1/Ref-1) secretion depends on estrogen receptor binding and intracellular calcium. (**A**) Effect of the competitive estrogen receptor (ER) inhibitor fulvestrant (100 nM for 2 h) on E2-induced APE1/Ref-1 secretion in cultured endothelial cells. (**B**) Effect of the endothelial nitric oxide synthase inhibitor N(ω)-nitro-L-arginine methyl ester (L-NAME) (10 mM for 1 h) on 17β-estradiol (E2)-induced APE1/Ref-1 secretion in cultured endothelial cells. (**C**) Effect of the cell permeable calcium chelator 1,2-Bis(2-aminophenoxy)ethane-N,N,N′,N′-tetraacetic acid tetrakis (acetoxymethyl ester) (BAPTA-AM) (10 µM for 30 min) on E2-induced APE1/Ref-1 secretion in cultured endothelial cells. Columns, mean (n = 4); dot plot, SE. *** *p* < 0.001, ** *p* < 0.01, and * *p* < 0.05 vs. Basal; ### *p* < 0.001, ## *p* < 0.01, and # *p* < 0.05 vs. E2 treated based on one-way ANOVA analysis followed by Bonferroni’s multiple comparison test.

The biological action of estrogen is mediated by estrogen receptor (ER) binding and its activation. Therefore, we evaluated whether E2 increased APE1/Ref-1 secretion via the ER (Figure 4A). Fulvestrant, a selective ER inhibitor, significantly reduced by about 80% of E2-induced APE1/Ref-1 secretion, compared to untreated cells (1.48 ± 0.09 ng/mL for E2 vs. 0.3 ± 0.09 ng/mL at 3 h for fulvestrant +E2, 4.14 ng/mL ± 0.42 ng/mL for E2 vs. 0.7 ± 0.27 ng/mL at 24 h for fulvestrant +E2). ER activation in vascular cells results in increased endothelial nitric oxide synthase (eNOS) activity [38]. As shown in Figure 4B, we attempted to determine whether E2-induced APE1/Ref-1 secretion was due to eNOS activity by comparing the results to those in the presence of the eNOS inhibitor L-NAME. However, pretreatment with L-NAME did not affect E2-induced increase of APE1/Ref-1 secretion in cultured endothelial cells.

It is also known that 17β-estradiol is involved in intracellular Ca^2+^ homeostasis in human endothelial cells [39]. To determine whether the APE1/Ref-1 secretion induced by E2 was caused by an increase in intracellular Ca^2+^ concentration, the cells were pretreated with the intracellular Ca^2+^ chelator BAPTA-AM and then evaluated. Interestingly, as shown in Figure 4C, pretreatment of vascular endothelial cells with BAPTA-AM significantly inhibited the APE1/Ref-1 secretion induced by E2 (2.87 ± 0.17 ng/mL for E2 vs. 0.7 ± 0.07 ng/mL at 3 h for BAPTA-AM + E2, 4.71 ± 0.9 ng/mL for E2 vs. 1.0 ± 0.18 ng/mL at 24 h for BAPTA-AM + E2). These findings suggest the binding of E2 to ER and increase in intracellular Ca^2+^ were major signaling processes required for E2-induced APE1/Ref-1 secretion in the cultured endothelial cells.

### 3.5. 17β-Estradiol-Induced APE1/Ref-1 Secretion Was Mediated through Exosomes

**Figure 5:**
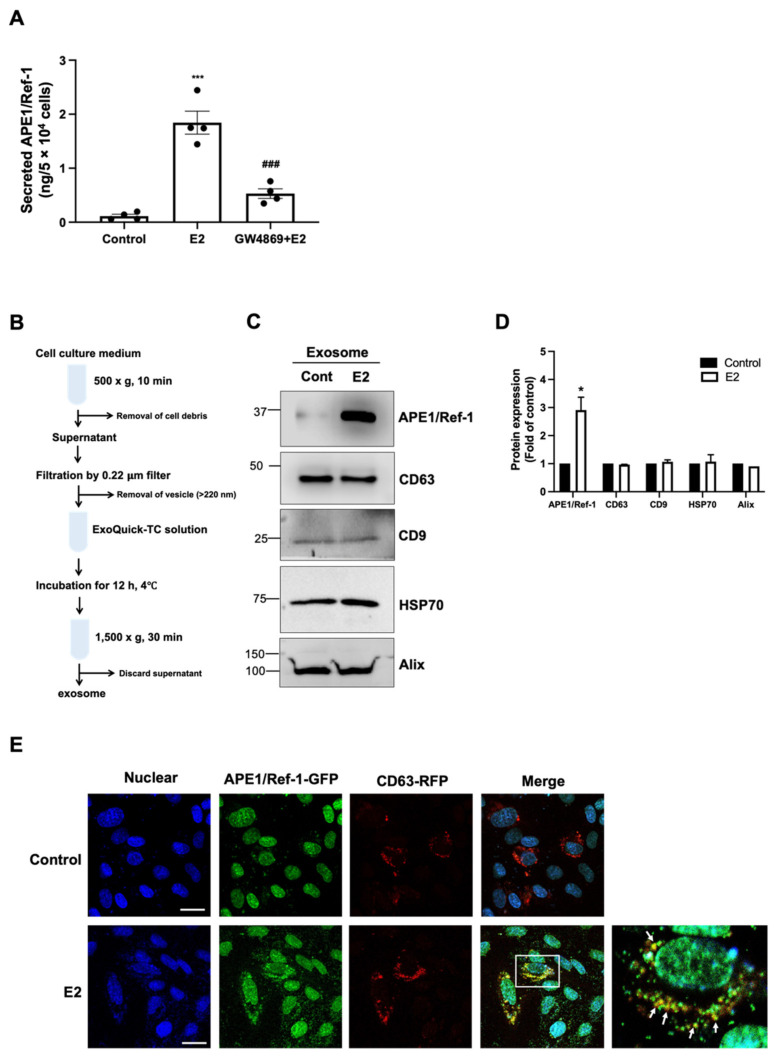
17β-estradiol(E2)-induced apurinic/apyrimidinic endonuclease-1/redox factor-1 (APE1/Ref-1) secretion is mediated through exosome pathway. (**A**) 17β-estradiol-induced APE1/Ref-1 secretion is inhibited by the exosome inhibitor GW4869 (5 µM). Columns, mean (n = 4); dot plot, SE. *** *p* < 0.001 and * *p* < 0.05 vs. control; ### *p* < 0.001 vs. E2-treated. (**B**) Schematic experimental steps for exosome isolation from the cultured medium of HUVECs. (**C**) 17β-estradiol (E2) increases APE1/Ref-1 expression in exosomes. Exosome isolation from culture media was confirmed with the presence of exosome-specific markers such as CD63, CD9, heat shock protein-70 (HSP70), and ALG-2 interacting protein X (Alix). (**D**) Summarized data of APE1/Ref-1 or exosome markers expression in exosomes. Columns, mean (n = 4); bar, SE. * *p* < 0.05 vs. control. Protein expressions are expressed as relative fold of control bands. (**E**) Immunofluorescence image in cultured endothelial cells transfected with plasmid *pAPE1/Ref-1-GFP* or *pCD63-RFP*. The images illustrate green fluorescent protein (GFP) fluorescence from APE1/Ref-1 and red fluorescent protein (RFP) fluorescence from CD63. The bottom right photo shows a 2.5× magnified image of the white box displayed in the merged image. Note, the cytoplasmic APE1/Ref-1 signals (green) are merged with the CD63 signals (red). Arrows indicate typical merged signal (orange). Scale bar showed 100 µm in length (white line).

Many cellular proteins lacking a signal peptide can be secreted through unconventional secretion processes, such as vesicle transport. Accordingly, we next investigated whether APE1/Ref-1 secretion induced by E2 occurred through the exosome pathway. The effect of the exosome inhibitor GW4869 was analyzed to determine whether E2-induced APE1/Ref-1 secretion is mediated with exosome pathway. As shown in Figure 5A, pretreatment of HUVECs with GW4869 significantly inhibited E2-induced APE1/Ref-1 secretion, suggested it was mediated through the exosome in endothelial cells.

We next investigated whether APE1/Ref-1 existed in exosome following E2 treatment. The exosome in the cell culture media was isolated as described with Material and Methods, and its experimental step was shown in Figure 5B. The successful exosome isolation from the culture media could be confirmed by the existence of exosome markers [40]. As shown in Figure 5C, exosome-specific markers such as CD63, CD9, HSP70, and Alix were expressed in isolated exosomes of cultured endothelial cells. A small amount of APE1/Ref-1 in basal condition was detected in exosomes, however, the exposure of E2 for 24 h in cultured endothelial cells was significantly increased approximately three-fold of APE1/Ref-1 in exosomes (Figure 5C,D). Interestingly, the expressions of exosome markers such as CD63, CD9, HSP70, and Alix were not changed by the exposure of E2.

Finally, we evaluated whether APE1/Ref-1 co-localized in vascular endothelial cell vesicles using immunofluorescence staining. As shown in Figure 5E, APE1/Ref-1 (green signal) was present in the nuclei of the cells while the exosome marker CD63 (red signal) was mainly present in the cytoplasm. However, when the cells were treated with E2 (100 pg/mL) for 3 h, the cytoplasmic expression of APE1/Ref-1 increased and co-localized with the CD63. The cytoplasmic APE1/Ref-1 and CD63 signals are merged to exhibit an orange color in the enlarged region of Figure 5E. Taken together, these data suggest that APE1/Ref-1 in response to E2 treatment was co-localized with CD63-positive exosome, suggesting APE/Ref-1 can be secreted through the exosome pathway in vascular endothelial cells.

## 4. Discussion

A significant finding of this study was the identification of an endogenous hormone that promotes APE1/Ref-1 secretion in vascular endothelial cells. We demonstrated that among the 12 hormones used, estrogen was the most potent in promoting the APE1/Ref-1 secretion.

APE1/Ref-1 has been detected in various biological solutions, including blood and urine [5,10,12,41,42,43]. In 2013, we reported that trichostatin A, a histone deacetylase inhibitor, increased the secretion of APE1/Ref-1 in HEK293 cells through intracellular hyperacetylation [4]. However, trichostatin A has been known to inhibit cell growth in cancer cells by inducing apoptosis [44], and even minimal cell death can alter the concentration of a specific protein in culture media, leading to doubts regarding the actual secretion of APE1/Ref-1. In the present study, we designed to exclude the possibility of passive APE1/Ref-1 release due to cell death, and to identify endogenous hormone that may actively increase APE1/Ref-1 secretion. Interestingly, our results showed that the endogenous hormones such as estrogen increased APE1/Ref-1 secretion without cell death as shown in Figure 1. Considering these results, we confirmed that actual secretion of APE1/Ref-1 in response to hormone.

In vascular cells, E2 binds to the ER, activates phosphoinositide 3-kinases (PI3K)/AKT signaling, stimulates eNOS, and consequently produces nitric oxide signaling [45,46]. In the present study, L-NAME and eNOS inhibitors did not directly affect E2-induced APE1/Ref-1 secretion in cultured endothelial cells. eNOS is constitutively expressed in vascular endothelial cells, and produces low nanomolar level of nitric oxide (NO) [47]. Therefore, low nanomolar level of NO did not affect APE1/Ref-1 secretion in endothelial cells. However, it is difficult to conclude that all nitric oxide concentrations or nitrosylation would not affect APE1/Ref-1 secretion. Previous study showed that plasma APE1/Ref-1 levels are increased in lipopolysaccharide-treated experimental animals [5]. Certain oxidative nitrogen-donating agents, such as S-nitrosoglutathione (GSNO) and S-nitroso-N-acetylpenicillamine (SNAP), promote nitrosylation by transferring their nitroxyl group to the protein thiol residue [48]. Previous reports have shown that GSNO selectively induces cytosolic translocation of APE1/Ref-1, where Cys93 and Cys310 are critical for nitrosylation-mediated cytosolic translocation [13].

Fulvestrant is a 7α-alkylsulphinyl analog of the 17β-estradiol structure and the first of the new type of ER antagonist that downregulates the receptor [49]. Fulvestrant competitively inhibits the binding of E2 to the ER and has a binding capacity of 89% compared to that of 17β-estradiol [50]. In the present study, fulvestrant effectively inhibited E2-induced APE1/Ref-1 secretion, suggesting that ER binding of estrogen is an important signal for APE1/Ref-1 secretion. In general, secretion pathways can be influenced by intracellular calcium, which is an important ion that regulates exocytosis and exosome release [39,51]. Cell membrane swelling and binding to other cell membranes are required during exosome formation and exocytosis, and Ca^2+^ is required for this process; therefore, it is likely that calcium may be required for the fusion events involved in exosome generation [51,52]. As APE1/Ref-1 secretion was completely inhibited by the calcium chelator BAPTA-AM, our findings indicate that APE1/Ref-1 secretion induced by E2 is dependent on intracellular calcium.

Recent studies have also demonstrated a great interest in exosomes as an important potential source of biomarkers as they participate in intercellular communication in cardiovascular disorders and tumors by transporting various proteins [53,54]. Proteins secreted via the classical secretory pathway require a secretory signal peptide or leader peptide [55,56]. However, computer-based analysis using SecretomeP predicts non-classical secretion of APE1/Ref-1 and indicates the absence of a putative N-terminal signal peptide [57]. Moreover, APE1/Ref-1 secretion is not blocked by brefeldin A, a typical inhibitor of the classical secretory pathway, again suggesting a non-classical pathway [16]. Secreted membrane-enclosed vesicles are collectively called extracellular vesicles and include exosomes and microvesicles.

Exosomes are typically 30–150 nm in diameter, and the size of the microvesicle range from 100 to 1000 nm in diameter [58]. In the present study, in order to minimize the unwanted mixing of microvesicles during exosome separation, pure exosomes were isolated using an Exoquick isolation kit that precipitates exosomes based on polyethylene glycol precipitation after removing microvesicle over 200 nm in diameter at 0.22 µm filter through filtration [59]. Exosome purification was confirmed by the expression of exosome-specific markers, CD63, CD9, HSP70, and Alix. Based on tetraspanins content, CD9 and CD63 were primarily used as classical exosome marker [60]. Exosome formation can be regulated by endosomal sorting complex required for transport proteins and its accessary protein such as Alix or heat shock protein-90 (HSP90) which is also used as exosome markers [40]. GW4869 is a neutral sphingomyelinase inhibitor and a commonly used pharmacological agent that inhibits exosome generation [61]. It blocks ceramide-mediated inward budding of multivesicular bodies and the release of mature exosomes from multivesicular bodies [62,63]. Our data showed that exosome inhibitor GW4869 significantly inhibited E2-induced APE1/Ref-1 secretion. APE1/Ref-1 secretion in CD63-positive exosomes was significantly increased by E2. These results suggest that APE1/Ref-1 secretion in response to E2 was mediated via exosomes in cultured endothelial cells.

Estrogen is a sex hormone responsible for the development of female reproductive systems and is produced primarily by the ovaries. At menopause, the ovarian follicles degenerate and circulating estrogen decreased to levels of castration. In postmenopausal women, a marked reduction of estrogen is risk factor of cardiovascular disease or osteoporosis [64,65,66]. In the present study, we also confirmed that reduced plasma APE1/Ref-1 levels in ovariectomized mice, administration of estrogen induced APE1/Ref-1 secretion in mice. These results suggested that low level of APE1/Ref-1 might be related with increased risks of cardiovascular disorders. Considering another aspect, abnormal production of female hormones or hormone-producing tumors can affect the level of APE1/Ref-1 in the blood. Therefore, when analyzing biomarkers such as APE1/Ref-1 in vascular inflammatory diseases, hormonal changes should also be considered.

## Figures and Tables

**Table 1:** Physiological plasma concentrations of hormones and treatment concentrations used in this study.

Hormone	Plasma Hormone Levels	Concentrations Used	Reference
Norepinephrine	0.3–0.7 μg/mL	16.1 μg/mL	[19]
Acetylcholine	0.036–0.584 μg/mL	14.6 μg/mL	[20]
Triiodothyronine (T3)	1.7–4.2 pg/mL	1000 pg/mL	[21]
Thyroxine (T4)	7–18 pg/mL	1000 pg/mL	[22]
Insulin-Like Growth Factor (IGF)	20–115 ng/mL	1000 ng/mL	[23]
Cortisol	29–250 ng/mL	1 μg/mL	[24]
Aldosterone	330–550 pg/mL	1000 pg/mL	[25]
Insulin	0.3–0.5 ng/mL	10 μg/mL	[26]
Glucagon	40–50 pg/mL	500 pg/mL	[27]
5α-Dihydrotestosterone (DHT)	0.4–6 ng/mL	100 ng/mL	[28]
	0.1–1 ng/mL		[29]
Progesterone (P4)	0.2–25 ng/mL	100 ng/mL	[30]
	1.5 ng/mL		[31]
Estrone (E1)	3–4 pg/mL	100pg/mL	[32]
	28.2 pg/mL		[33]
17β-estradiol (E2)	0.5–9 pg/mL	100 pg/mL	[34]
	0.6–7.1 pg/mL		[35]
Estriol (E3)	<200 pg/mL	500 pg/mL	[36]
	7.9–11.1 pg/mL		[37]
